# Reduced transforming growth factor-beta signaling in cartilage of old mice: role in impaired repair capacity

**DOI:** 10.1186/ar1833

**Published:** 2005-09-30

**Authors:** EN Blaney Davidson, A Scharstuhl, EL Vitters, PM van der Kraan, WB van den Berg

**Affiliations:** 1 Experimental Rheumatology and Advanced Therapeutics, St Radboud University Medical Centre Nijmegen, Geert Grooteplein 26, 6525 GA Nijmegen, The Netherlands

## Abstract

Osteoarthritis (OA) is a common joint disease, mainly effecting the elderly population. The cause of OA seems to be an imbalance in catabolic and anabolic factors that develops with age. IL-1 is a catabolic factor known to induce cartilage damage, and transforming growth factor (TGF)-beta is an anabolic factor that can counteract many IL-1-induced effects. In old mice, we observed reduced responsiveness to TGF-beta-induced IL-1 counteraction. We investigated whether expression of TGF-beta and its signaling molecules altered with age. To mimic the TGF-beta deprived conditions in aged mice, we assessed the functional consequence of TGF-beta blocking. We isolated knee joints of mice aged 5 months or 2 years, half of which were exposed to IL-1 by intra-articular injection 24 h prior to knee joint isolation. Immunohistochemistry was performed, staining for TGF-beta1, -2 or -3, TGF-betaRI or -RII, Smad2, -3, -4, -6 and -7 and Smad-2P. The percentage of cells staining positive was determined in tibial cartilage. To mimic the lack of TGF-beta signaling in old mice, young mice were injected with IL-1 and after 2 days Ad-LAP (TGF-beta inhibitor) or a control virus were injected. Proteoglycan (PG) synthesis (^35^S-sulfate incorporation) and PG content of the cartilage were determined. Our experiments revealed that TGF-beta2 and -3 expression decreased with age, as did the TGF-beta receptors. Although the number of cells positive for the Smad proteins was not altered, the number of cells expressing Smad2P strongly dropped in old mice. IL-1 did not alter the expression patterns. We mimicked the lack of TGF-beta signaling in old mice by TGF-beta inhibition with LAP. This resulted in a reduced level of PG synthesis and aggravation of PG depletion. The limited response of old mice to TGF-beta induced-IL-1 counteraction is not due to a diminished level of intracellular signaling molecules or an upregulation of intracellular inhibitors, but is likely due to an intrinsic absence of sufficient TGF-beta receptor expression. Blocking TGF-beta distorted the natural repair response after IL-1 injection. In conclusion, TGF-beta appears to play an important role in repair of cartilage and a lack of TGF-beta responsiveness in old mice might be at the root of OA development.

## Introduction

Osteoarthritis (OA) is a common joint disease characterized by cartilage damage, osteophyte formation and thickening of the joint capsule. The etiology of OA is unknown, but OA is strongly correlated with age. OA may be a result of an age-related alteration in responsiveness of cells to anabolic and catabolic stimuli.

IL-1 is a cytokine that plays an important catabolic role in OA. IL-1 is highly expressed by chondrocytes of joints that are affected by OA, both in mice and humans [1,2]. Patients with OA have high levels of IL-1 in their synovial fluids as well [3]. IL-1 itself can induce cartilage damage [4] by reducing proteoglycan (PG) synthesis, increasing matrix metalloproteinase expression [5], and stimulating nitric oxide production [6].

Transforming growth factor (TGF)-beta is an important anabolic factor in OA. It is very beneficial for cartilage as it stimulates PG and collagen type II synthesis and can downregulate cartilage-degrading enzymes [7-13]. In addition, TGF-beta is able to counteract IL-1 induced suppression of PG synthesis [9,14-16]. Through this action TGF-beta is able to protect cartilage from damage by IL-1 [9,17,18]. In humans, expression of an asporin variant with a high TGF-beta inhibitory effect is significantly correlated with an increased incidence of OA [19].

Old animals show more prolonged suppression of PG synthesis after IL-1 exposure than young mice [4] and display a reduced response to counteraction of IL-1 by TGF-beta [20]. This indicates a shift in response to catabolic and anabolic stimuli, eventually leading to loss of cartilage homeostasis and OA.

TGF-beta signals predominantly through two receptors, TGF-beta-RI (ALK5) and TGF-beta-RII. TGF-beta binds to the type II receptor, recruits and phosphorylates the type I receptor and subsequently activates its receptor Smad, Smad2 or Smad3, by phosphorylation [21]. Thereafter, the phosphorylated Smad2 or Smad3 forms a complex with the common-Smad, Smad4. The complex is subsequently translocated to the nucleus where TGF-beta responsive genes are transcribed [22]. Inside the cell there are also inhibitory Smads (Smad6 and Smad7) that can prevent TGF-beta signaling [23,24].

We postulate that the lack of responsiveness to TGF-beta counteraction of IL-1 in old mice is due to an overall lack of responsiveness to TGF-beta caused by a down regulation of receptors and/or Smad expression or and increase in inhibitory Smads. Therefore, we investigated the expression of the various TGF-betas (1, 2 and 3) as well as their signaling molecules (TGF-beta-RI and TGF-beta-RII, Smad2, Smad-2P, Smad3, Smad4, Smad6 and Smad7) immunohistochemically in the cartilage of knee joints of young and old mice. In addition, we assessed whether these expression levels were altered differently in young and old mice by intra-articular injection of IL-1α.

We show that old mice have a profoundly lower expression of TGF-beta receptors (I and II) than young mice, which correlates with less Smad-2 phosphorylation. IL-1 itself had little effect on the expression of TGF-beta signaling molecules in cartilage.

To investigate whether reduced TGF-beta response could cause the reduced repair capacity in old mice, we mimicked the lack of TGF-beta by blocking TGF-beta activity with latency associated peptide (LAP) after IL-1 insult. This demonstrated that endogenous TGF-beta was required for a normal repair response and that lack thereof aggravates cartilage damage.

## Materials and methods

### Animals

Male C57Bl/6 mice aged 5 months or 2 years were used. Animals were kept in filtertop cages with woodchip bedding under standard pathogen free conditions. They were fed a standard diet with tap water *ad libitum*. The local animal committee approved this study.

### Experimental design

TGF-beta counteraction of IL-1 effects is most likely mediated by TGF-betaRI, TGF-betaRII and the intercellular Smad proteins. We investigated if young (n = 14) and old (n = 14) mice differ in expression of these TGF-beta signaling mediators. Therefore, knee joints were isolated and prepared for immunohistochemistry. Half of the joints were prepared for paraffin sections, half were prepared for frozen sections. The number of cells staining positive for the various proteins were measured with a computerized imaging system. In addition to the comparison between young and old mice, we checked whether IL-1α injection 24 h prior to knee joint isolation (10 ng) (R&D Systems, Wiesbaden, Germany) influenced the expression patterns. Thus, the right knee joint of every mouse was injected with IL-1α and the left knee served as the non-injected group.

To assess whether lack of TGF-beta could indeed cause reduced repair capacity in old mice, young mice (n = 14) were injected intra-articularly with IL-1. Two days later we injected an adenovirus over-expressing the TGF-beta inhibitor LAP [25]. This inhibitor scavenges endogenous TGF-beta in the synovial fluid, preventing it from binding to its receptor. After 4 days, patellae were isolated for measurement of PG synthesis or whole knee joints were isolated for histology to measure PG content in the cartilage.

### Histology

For the different classes of Smads, knee joints were decalcified for 14 days in EDTA/PVP and subsequently cryosections of total knee joints (7 μM) were prepared and stored at -20°C. Before use, sections were air-dried for 30 minutes and freshly prepared paraformaldehyde (4%, 5 minutes) was used to fix the sections.

Immunohistochemistry for TGF-beta1, TGF-beta2, TGF-beta3, TGF-betaRI, TGF-betaRII and Smad-2P, as well as Safranin O/Fast Green staining, were performed on paraffin sections from total knee joints. Knee joints were fixed in phosphate buffered formalin for 7 days. They were dehydrated using an automated tissue-processing apparatus (Tissue Tek VIP, Sakura, Ramsey, MN, USA) and embedded in paraffin. Tissue sections of 7 μM were prepared.

### Immunohistochemistry

Sections were deparaffinized and washed with PBS. For antigen unmasking, sections were incubated in citrate buffer (0.1 M sodiumcitrate, 0.1 M citric acid) for 2 h. Endogenous peroxidase was blocked with 1% hydrogen peroxidase in methanol for 30 minutes. Thereafter, sections were blocked with 5% normal serum of the species in which the secondary antibody was produced. Specific primary antibodies against TGF-beta1, TGF-beta2, and TGF-beta3 (1.0 μg/ml), TGF-betaRI and TGF-betaRII, Smad2, Smad3 and Smad4 (0.5 μg/ml), Smad6 (1.0 μg/ml), Smad7 (3.3 μg/ml) and Smad-2P (1:100) were incubated overnight at 4°C. (Smad6 antibody was purchased from Invitrogen (Breda, The Netherlands), Smad-2P from Cell Signaling Technology (Beverly, MA, USA), and all other primary antibodies were purchased from Santa Cruz Biotechnology Inc (Santa Cruz, CA, USA)). As a negative control, the primary antibody was replaced with goat or rabbit IgGs. After extensive washing in PBS, the appropriate biotin labeled secondary antibody was used at a concentration of 2 μg/ml in 1% bovine serum albumin/PBS for 2 h (Vector Laboratories Inc, Burlingame, CA, USA), followed by a biotin-streptavidine detection system according to the manufacturer's protocol (Vector Laboratories Inc.). Bound complexes were visualized via reaction with 3',3'-diaminobenzidine (Sigma Chemicals Co, Zwijndrecht, The Netherlands) and H_2_O_2 _resulting in a brown precipitate. Sections were briefly counterstained with hematoxylin and mounted with Permount.

### Image analysis: quantification of positively stained articular chondrocytes

For the different antigens, the number of positive articular chondrocytes in the tibia was determined by a blinded observer. The microscopic image was displayed on a computer monitor using the Qwin image analysis system (Leica Imaging Systems, Rijswijk, The Netherlands) and a Leica DC 300F digital camera. The area representing the non-calcified articular cartilage was selected by hand. For each antigen, a threshold was set in such a manner that only chondrocytes that were found to be positive (brown stained cell) as judged by the observer were selected. The computer program determined the number of positive cells in the cartilage for the different antigens. For each knee joint, the expression of the different antigens was measured in at least three tissue sections. The intensity of the staining was not taken into account as no obvious differences were observed in staining intensities in the different experimental groups: young/old or -IL-1/+IL-1. The obtained values were averaged and the average per treatment group was determined. To correct for differences in cell number between young and old mice, the average number of chondrocytes in the non-calcified cartilage was determined in sections stained with hematoxylin only for both paraffin and frozen sections. This was based on a similar selection procedure to that described above with the exception that selection of chondrocytes was based on the blue staining from heamatoxylin instead of brown staining. The average number of chondrocytes per sections was calculated for every joint.

### Image analysis: proteoglycan content

PG content of articular cartilage was measured in sections stained with Safranin O and Fast Green using a computerized imaging system as previously described [25]. Briefly, Safranin O stains PGs in the cartilage red. A blinded observer captured an image on screen and selected the cartilage. The computer then measured the amount of blue light passing through the selected area. The higher the amount of light passing through, the lower the amount of PGs in cartilage. The average of three sections per knee joint was calculated.

### Proteoglycan synthesis

PG synthesis was assessed by measurement of ^35^S-sulfate incorporation. Isolated patellae were immediately placed in Dulbecco's modified Eagle's medium with gentamicin (50 mg/ml) and pyruvate. After half an hour, this medium was replaced by medium containing ^35^S-sulfate 20 μCi/ml in which patellae were incubated for 3 h at 37°C and 5% CO_2_. Thereafter, patellae were further prepared for determining the amount of ^35^S-sulfate incorporation in the articular cartilage as previously described [22].

### Statistical analysis

Results were analyzed with the Student's t-test and considered significant if the p-value was smaller than 0.05.

## Results

### Chondrocyte cell number is reduced with age

**Figure 1:**
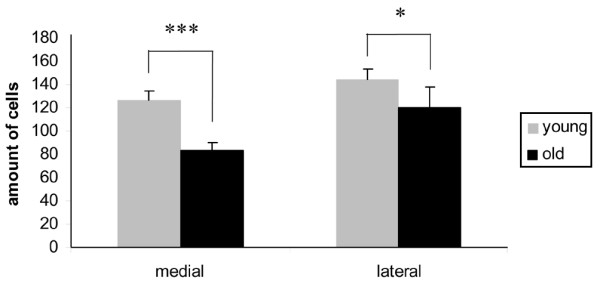
Number of cells in medial and lateral tibial cartilage of 5 month and 2 year old C57Bl/6 mice. Paraffin sections of knee joints of young (5 months old) and old (2 years old) mice were stained with hematoxylin and eosin after which a blinded observer used a computerized imaging system to count the number of chondrocytes in the tibial cartilage. Old mice have a lower number of cells in their cartilage than young mice. The reduction in cell number is more pronounced on the medial side of the joint. Error bars display the standard error. For statistical analysis, a Student's t-test was used. * = p < 0.05; ** = p < 0.005; *** = p < 0.0005.

The percentage of cells expressing the different TGF-beta signaling proteins in murine cartilage was calculated by correction for the total number of cells present in the articular cartilage of the tibia. Therefore, the total number of cells in both medial and lateral tibial cartilage was quantified for all experimental groups by computerized quantification of cell number in hematoxyline and eosin (H&E) stained sections. Old mice had a significantly lower number of chondrocytes in the tibial cartilage: the reduction was more pronounced in the medial tibial cartilage, with a reduction in cell number of 34%; the number of cells in lateral tibial cartilage had reduced 17%. (Fig. 1). Treatment with IL-1 had no effect on the total number of cells (data not shown).

### Reduction of various TGF-beta signaling molecules with age

**Figure 2:**
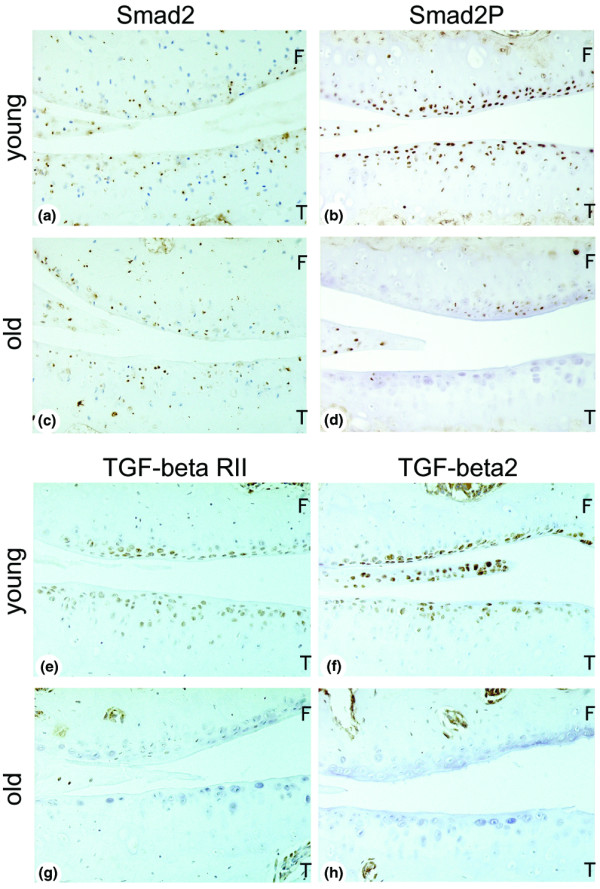
Staining of various transforming growth factor (TGF)-beta signaling molecules in cartilage. Paraffin sections of knee joints of young and old mice were stained with antibodies against **(a,c) **Smad2, **(b,d) **Smad-2P, **(e,g) **TGF-beta receptor II (TGF-betaRII) and **(f,h) **TGF-beta2. The medial tibia of the young mice clearly show a high number of cells staining positive for (b) Smad-2P, (e) TGF-betaRII and **(f) **TGF-beta2, whereas the **(d,g,h) **old mice had only a very low number of cells staining positive for these factors. (a,c) Smad2 staining remained unchanged with age. F, femur; T, tibia.

**Figure 3:**
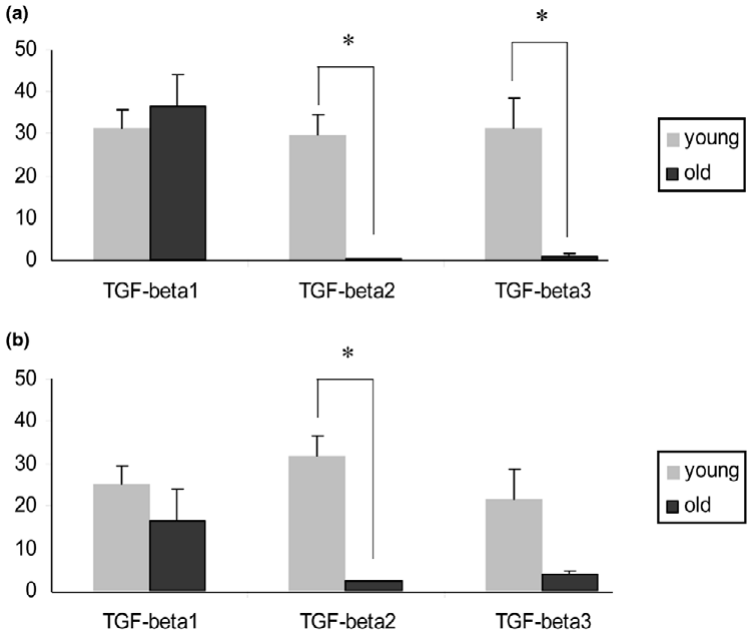
Percentage of cells expressing transforming growth factor (TGF)-beta in medial and lateral tibial cartilage. Paraffin sections of knee joints of young (5 months old) and old (2 years old) mice were stained immunohistochemically with antibodies against TGF-beta1, TGF-beta2 or TGF-beta3. Subsequently, the number of cells staining positive in the cartilage were scored with a computerized imaging system and corrected for the total number of cells. **(a) **In medial cartilage, TGF-beta2 and TGF-beta3 expression were significantly reduced with age. **(b) **In lateral cartilage, TGF-beta2 was significantly reduced. Error bars display the standard error. For statistical analysis, a Student's t-test was used. * = p < 0.05.

To assess whether the reduced TGF-beta responsiveness in old mice was due to a lower amount of TGF-beta expression we compared the number of TGF-beta positive cells in the tibial cartilage of young (5 months old) and old mice (2 years old) in immunohistochemically stained sections. The number of positive cells was quantified with a computerized imaging system and corrected for the total amount of chondrocytes. In both medial and lateral tibial cartilage, age had no effect on the number of TGF-beta1 expressing cells. However, the number of cells expressing TGF-beta2 in old mice had reduced from 30% to almost no positive cells left (average of 0.2%) on the medial side of the joint and from 32% to 2% on the lateral tibial cartilage (Fig. 2f, h). TGF-beta3 showed a similar pattern in medial tibial cartilage, where the number of positive cells was 31% in young mice compared to 1% in old mice. On the lateral side of the joint, ageing also resulted in a lower number of TGF-beta3 positive cells, but this was not significant (Fig. 3).

**Figure 4:**
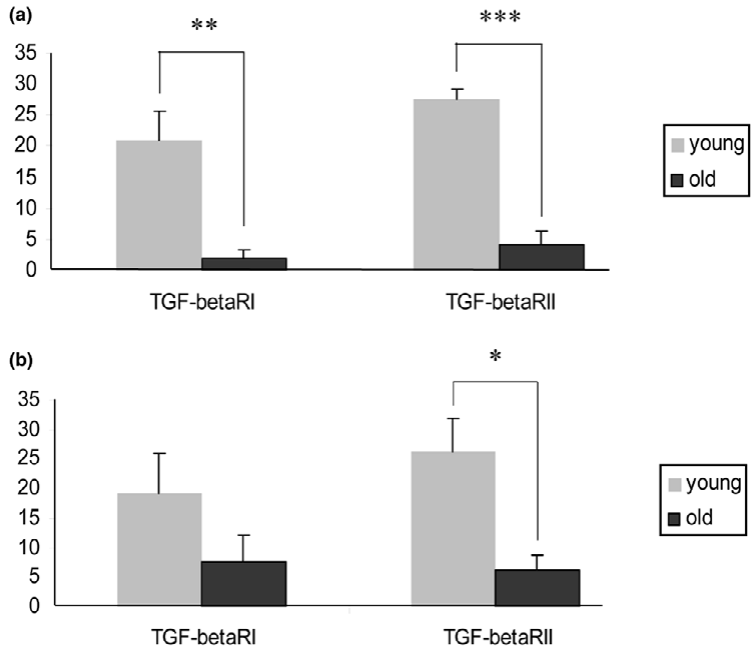
Percentage of cells expressing transforming growth factor (TGF)-beta receptors (TGF-betaRs). Paraffin sections of knee joints of young (5 months old) and old mice (2 years old) were stained immunohistochemically with antibodies against TGF-betaRI or TGF-betaRII. Subsequently, the number of cells staining positive in the cartilage were scored with a computerized imaging system and corrected for the total number of cells. The expression of both receptors was reduced with age in both **(a) **medial and **(b) **lateral tibial cartilage, but the reduced TGF-betaRII was significant only in lateral tibial cartilage. Error bars display the standard error. For statistical analysis, a Student's t-test was used. * = p < 0.05; ** = p < 0.005; *** = p < 0.0005.

We also examined the effect of aging on the number of cells staining positive for the TGF-betaRs. TGF-betaRI was expressed by a significantly lower number of cells in the medial tibial cartilage in old mice compared to young mice, 2% compared to 21%, respectively. On the lateral side, the number of TGF-betaRI positive cells was also lower in old mice, but this was not significant. The amount of cells expressing TGF-betaRII was significantly lower in old mice, both on the medial and on the lateral side of the joint. On the medial side, the number of immunopositive cells was reduced with age from 27% in young mice to 4% in old mice; in the lateral tibial cartilage the reduction was from 26% to 6% (Figs 4 and 2e, g).

**Figure 5:**
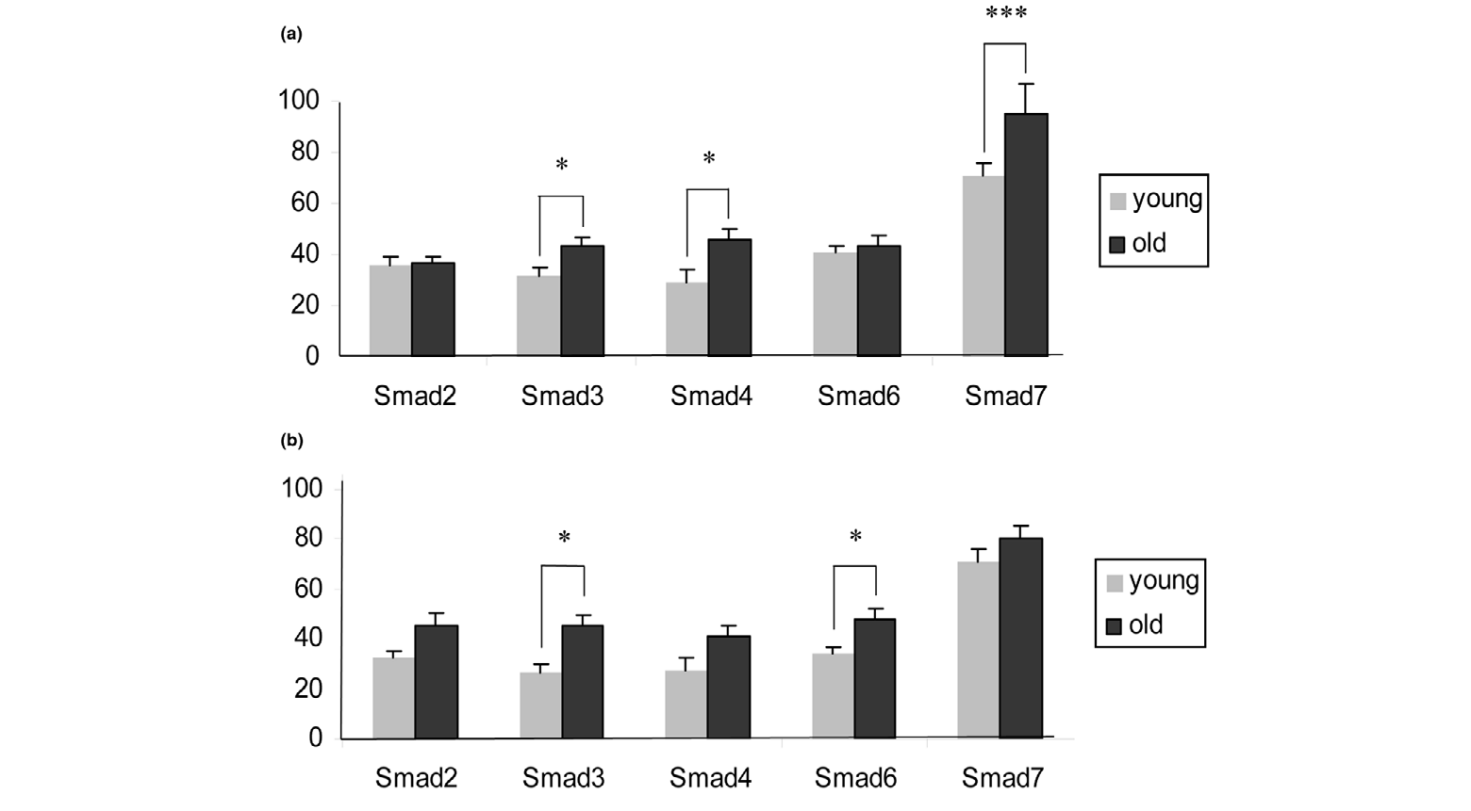
Percentage of cells expressing Smad in medial and lateral tibial cartilage. Frozen sections of knee joints of young (5 months old) and old (2 years old) mice were stained immunohistochemically with antibodies against Smad2, Smad3, Smad4, Smad6 or Smad7. Subsequently, the number of cells staining positive were scored with a computerized imaging system and corrected for the total number of cells. **(a) **In medial tibial cartilage, expression of Smad3, Smad4 and Smad7 increased with age. **(b) **In lateral tibial cartilage Smad3 and Smad6 expression increased with age. Error bars display the standard error. For statistical analysis, a Student's t-test was used. * = p < 0.05; *** = p < 0.0005.

In contrast to the receptors, the number of cells positive for the several Smad molecules had hardly changed with age. The percentage of cells positive for receptor-Smad Smad2 was equal in young and old mice (Fig. 2a, c). The expression of receptor-Smad Smad3 had increased in old mice as well as the percentage of cells positive for the common-Smad, Smad4, but only in the medial tibial cartilage. The inhibitory Smad, Smad6, was not altered with age in the medial tibial cartilage, but was higher in old mice on the lateral side. Smad 7 was significantly higher in old mice, but this was limited to the medial tibial cartilage (Fig. 5).

**Figure 6:**
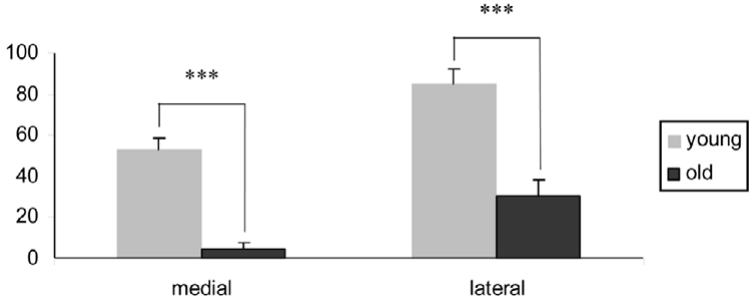
Percentage of cells expressing Smad-2P in medial and lateral tibial cartilage. Paraffin sections of knee joints of young (5 months old) and old (2 years old) mice were stained immunohistochemically with antibodies against Smad-2P. Subsequently, the number of cells staining positive were scored with a computerized imaging system and corrected for the total number of cells. The Smad-2P expression was reduced with age in both medial and lateral tibial cartilage. Error bars display the standard error. For statistical analysis, a Student's t-test was used. *** = p < 0.0005.

Despite the lack of difference in Smad2 expression between young and old mice, the phosphorylated variant of this Smad, Smad-2P, was significantly reduced in old mice in both medial and tibial cartilage. In the medial tibial cartilage, the percentage of cells staining positive for Smad-2P was 53% in young mice compared to 5% in old mice. On the lateral side, aging had lowered the amount of immunopositive cells from 85% to 30% (Figs 6 and 2b, d). This indicates a decrease in active TGF-beta signaling in old mice, possibly related to the decreased number of TGF-betaRs in old mice.

**Figure 7:**
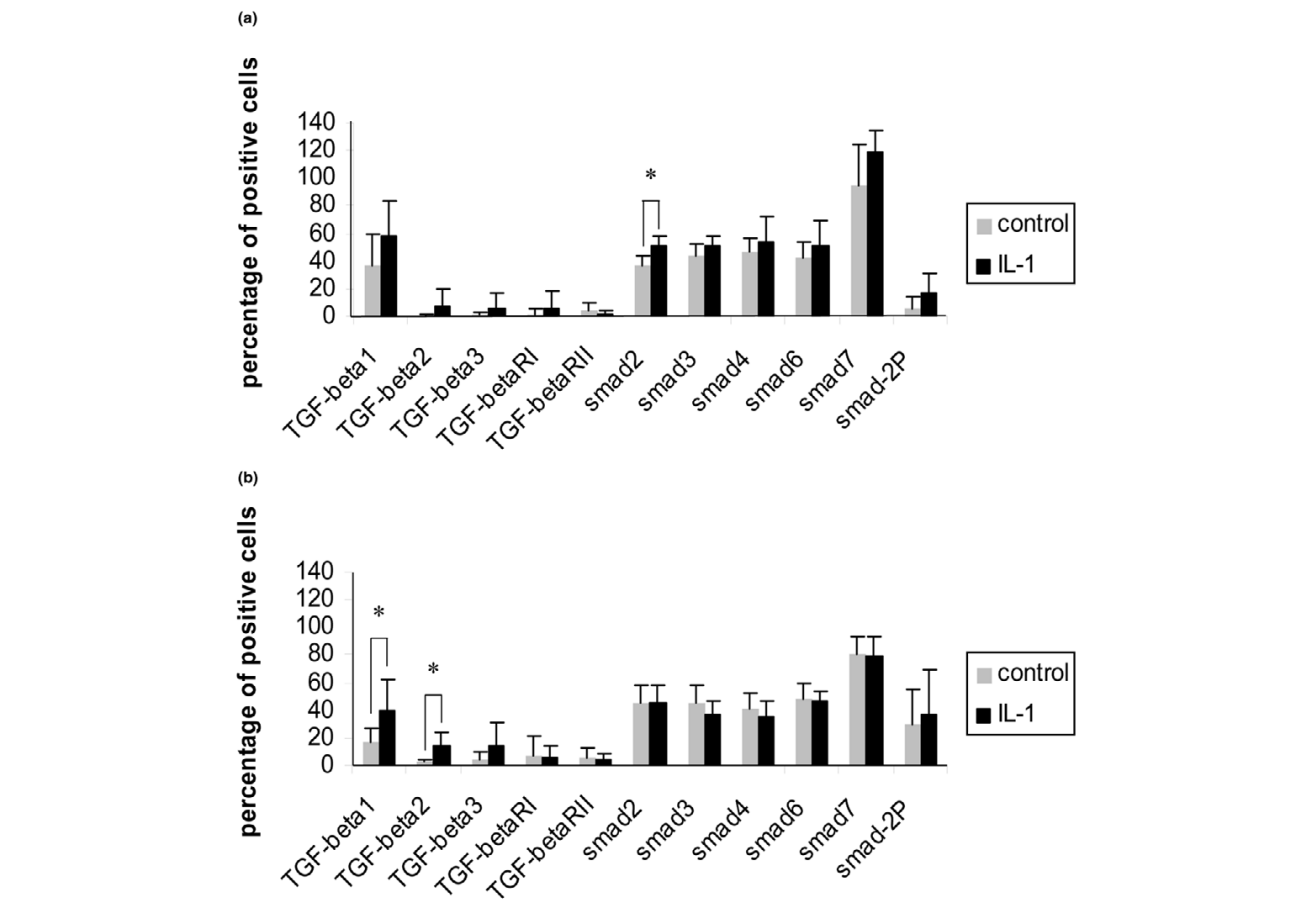
Effect of IL-1 on expression of TGF-beta signaling proteins in cartilage. Knee joints of **(a) **young (5 months old) and **(b) **old (2 years old) mice were injected with IL-1 24 h prior to isolation of the knee joints. Paraffin sections of knee joints were stained immunohistochemically for TGF-beta1, TGF-beta2, TGF-beta3, TGF-beta receptor I (TGF-betaRI), TGF-betaRII, Smad2, Smad3, Smad4, Smad6, Smad7 and Smad-2P. Subsequently, the number of cells staining positive were scored with a computerized imaging system and corrected for the total number of cells. After IL-1 injection, Smad2 expression increased only in the medial tibial cartilage and TGF-beta1 and TGF-beta2 expression increased only in the lateral tibial cartilage. Error bars display the standard error. For statistical analysis, a Student's t-test was used. * = p < 0.05.

To assess whether IL-1 itself altered TGF-beta signaling in old mice, thereby reducing the counteractive abilities of TGF-beta to IL-1, we examined the effect of IL-1 injection on the expression of TGF-beta signaling components in the articular cartilage. Injection of IL-1 24 h prior to knee joint isolation resulted in an increased expression of TGF-beta1 and TGF-beta2 in lateral tibial cartilage in old mice and a higher number of Smad2 positive cells in the medial tibial cartilage. IL-1 treatment did not influence TGF-beta receptor, Smad or Smad2P expression in old mice (Fig. 7). IL-1 did not alter the expression of the TGF-beta signaling components in young mice.

### Effect of blocking TGF-beta on proteoglycan synthesis and proteoglycan content

**Figure 8:**
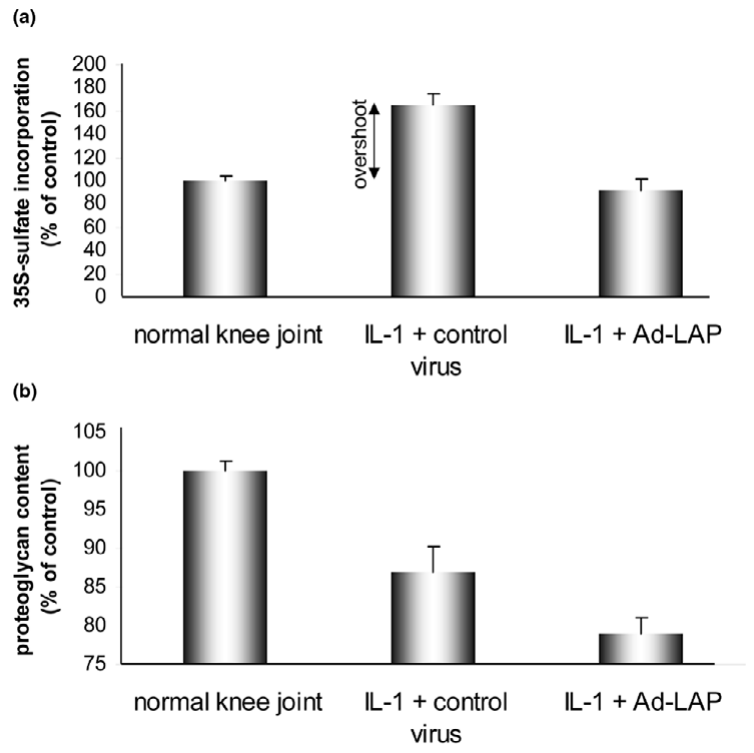
Effect of transforming growth factor (TGF)-beta deprivation on intrinsic cartilage repair capacity. Murine knee joints of young mice were injected with IL-1. After two days an adenovirus expressing the TGF-beta inhibitor latency associated peptide (LAP) was injected intra-articularly. Four days after the initial injections with IL-1, patellae were isolated for ^35^S-sulfate incorporation and whole knee joints were isolated for histology. **(a) **^35^S-sulfate incorporation into isolated patellar cartilage after treatment with IL-1 and Ad-LAP. IL-1 treatment induces an initial decrease in ^35^S-sulfate incorporation, but by day 4 the ^35^S-sulfate incorporation increased above normal levels, indicating an overshoot in proteoglycan synthesis. By blocking endogenous TGF-beta with LAP, this overshoot is completely abolished. **(b) **Proteoglycan content of patellar cartilage after treatment with IL-1 and Ad-LAP. IL-1 injection results in depletion of proteoglycans in cartilage. Blocking endogenous TGF-beta with LAP results in an aggravation of this depletion beyond IL-1 induced damage alone.

To assess the functional consequence of depressed TGF-beta signaling, we blocked TGF-beta by adenoviral overexpression of the TGF-beta inhibitor LAP two days after IL-1 insult. Four days after primary insult, knee joints were isolated for assessment of PG synthesis and PG content. PG synthesis was measured by ^35^S-sulfate incorporation into cartilage *ex vivo*. A normal response to IL-1 insult is an initial drop in PG synthesis the first 2 days after IL-1 injection, followed by a rapid increase in synthesis within the next 2 days [4]. The increased synthesis levels are above normal turnover levels. LAP over-expression after IL-1 injection was able to completely block this intrinsic increase in PG synthesis as shown by the ^35^S-sulfate incorporation, which was lower than after IL-1 insult alone (Fig. 8a).

In addition, PG content was measured by quantification of Safranin O staining intensity of the cartilage. The block of endogenous TGF-beta resulted in an aggravation of cartilage damage as the PG content of the cartilage was significantly reduced beyond IL-1 induced PG depletion (Fig. 8b). These data show that deprivation of TGF-beta resulted in a reduced repair capacity of the cartilage.

## Discussion

OA is characterized by cartilage damage with an increasing incidence with age. The etiology of OA is unknown, but an imbalance between catabolic and anabolic factors appears to be involved. Whereas chondrocytes of young mice respond well to TGF-beta counteraction of IL-1, those of old mice show less efficient counteracting of IL-1 by TGF-beta [20]. In addition, they display prolonged suppression of PG synthesis. This might be due to a decreased response to TGF-beta in cartilage of old mice. We compared, therefore, the expression of TGF-beta and the TGF-beta signaling components in cartilage of young and old mice. The cartilage of old mice contained a lower number of cells than young mice. We thus corrected our findings for the total number of cells in the examined cartilage. In this study, only the tibial cartilage is discussed, but similar changes occurred in the femoral cartilage. The reduced cell number we found in old mice corresponds to the decreased number of cells that was found in cartilage of human donors older than 40 [26]. A decrease in chondrocyte cell number could be due to an age-related decline in (TGF-beta-induced) chondrocyte proliferation rate [27,28].

Our results show that old mice have significantly lower numbers of cells expressing TGF-beta2 and TGF-beta3 than young mice. In addition, old animals had a significantly lower number of chondrocytes expressing TGF-betaRs. The lack of responsiveness to TGF-beta counteraction in old mice is not likely a result of alterations in Smad expression, as they are unaffected or even elevated by aging. Smad3 was elevated in tibial cartilage, and in the medial tibial cartilage we found an elevation of Smad4 with age. The basal material for signaling inside the cell is present, only the action is lacking. This lack of action might be due to the reduced receptor expression combined with a drop in TGF-beta2 and TGF-beta3 in old mice. This could also explain the lower Smad2 phosphorylation in old mice. Smad2 itself is not a problem as it is present in equal numbers in both young and old mice, but if there are less receptors and less ligands, Smads are unlikely to be phosphorylated in high amounts.

In lateral tibial cartilage we found an elevation of Smad6 expression with age, while in medial tibial cartilage Smad7 was elevated with age; these changes were restricted to one cartilage surface only instead of both. Although it might contribute, it is unlikely that this elevation is the cause of the overall unresponsiveness to TGF-beta.

We wanted to make sure that IL-1 itself did not alter TGF-beta signaling and cause the reduced counteraction. Therefore, mice were exposed to IL-1 prior to knee joint isolation. IL-1 treatment had only little effect on TGF-beta signaling. In old mice, we found an upregulation of TGF-beta1 and TGF-beta2 in lateral tibial cartilage. In the medial tibial cartilage, we observed an IL-1-induced increase in Smad2. Although there was elevation of these factors, it had no effect on Smad-2P, indicating that IL-1 treatment did not alter the outcome of TGF-beta signaling.

Iqbal *et al*. [29] found a decrease in the expression of mRNA for TGF-beta1, TGF-beta2 and TGF-beta3 with age in equine cartilage, supporting our findings. It is not clear why the TGF-beta isoforms show a different pattern but it is known that all three isoforms are differentially regulated and have a different promotor region. Also, during embryogenis all three isoforms show a different, developmental stage related expression pattern [30]. Gómez-Camarillo *et al*. [31] also showed a progressive decrease of TGF-betaRI with age. Matsunaga *et al. *[32] found similar expression patterns in cervical intervertebral discs in mice. They showed a decrease in expression of TGF-beta1, TGF-beta2 and TGF-beta3 as well as TGF-betaRI and TGF-betaRII with age. In myogenic progenitor cells in mice, Beggs *et al*. [33] described similar observations. They found that TGF-betaRI and TGF-betaRII were downregulated and Smad2, Smad3, Smad4 and Smad7 remained unchanged [33]. These data indicate that our findings are similar to those found in other species and cell types and that the phenomenon of reduced TGF-betaRs and reduced TGF-beta expression it is not limited to cartilage of murine knee joints.

IL-1 treatment increased the expression of TGF-beta1 and TGF-beta2 in tibial cartilage. Andriamanalijaona *et al*. [34] have also shown the ability of IL-1 to increase TGF-beta1 of articular chondrocytes. Kaiser *et al*. [35] showed that IL-1 treatment resulted in elevated expression of Smad7 mRNA *in vitro *after 3 days. In our *in vivo *experiment, however, no significant alterations in inhibitory Smad expression were found. In contrast to Kaiser *et al*. [35], we measured the percentage of cells expressing Smad7 one day after IL-1 injection *in vivo*. The discrepancies in time, measurement and system probably explain why different results were found.

We previously examined TGF-beta expression in OA. In severe OA in STR/ort mice, we did not find any TGF-beta expression or Smad-2P at all, whereas younger STR/ort mice with only mild damage still expressed both factors (data not shown). In addition, others have also found discrepancies between OA cartilage and healthy cartilage with respect to TGF-beta expression. Gomez-Camarillo and Kouri [31] showed that TGF-beta1 receptors were very scarce in experimental OA. The drop in expression levels of TGF-beta and their signaling molecules that we found in old mice might precede OA.

The expression patterns in the cartilage suggest that a lack of TGF-beta signaling plays a potential role in the reduced repair capacity in old mice and possibly in OA. To further investigate whether the disturbed TGF-beta signaling could cause a reduction in repair, we inhibited endogenous TGF-beta after IL-1 insult. This resulted in a total block of the increased PG synthesis, thereby reducing the intrinsic repair capacity of the cartilage. The reduced PG synthesis resulted in an aggravation of the IL-1-induced PG loss in cartilage. These results show that not only do old mice have a reduced TGF-beta signaling capacity, but also that disrupted TGF-beta signaling can indeed induce a distorted repair capacity of cartilage.

It has been hypothesized that TGF-beta treatment can be used as a factor for cartilage repair. However, old mice respond poorly to TGF-beta, so the use of TGF-beta for repair might be more difficult than expected. It has already been shown that human articular chondrocytes stimulated with TGF-beta1, fibroblast growth factor-2 and platelet derived growth factor-BB, contained more glycosaminoglycans than non-stimulated controls, but only if donors were younger than 40 [26]. In addition, stimulation of equine articular cartilage with TGF-beta resulted in lower [^35^S]Na_2_SO_4 _incorporation in horses of 20 years old compared to 9 month old horses [26,29]. Although the response to TGF-beta is reduced with age, it does not mean that the cartilage does not respond at all. There was still an increase in incorporation of [^35^S]Na_2_SO_4 _after TGF-beta stimulation found by Livne *et al*. [36] in mice, but it has to be considered that this response in old animals cannot be compared to the massive stimulation in young animals. However, finding ways to stimulate cartilage repair bypassing the TGF-beta receptor pathway appears to be an attractive option to boost repair of aged cartilage.

## Conclusion

Our data show that there are less chondrocytes expressing TGF-betaRs in cartilage in old mice. Smad expression is unchanged, but Smad2 phosphorylation is reduced with age. These data suggest that the reduced TGF-beta counteraction of IL-1 induced cartilage damage of old mice is due to an overall lack in TGF-beta signaling capacity. Blocking endogenous TGF-beta in young mice induced a distorted repair capacity in cartilage. The reduced ability of chondrocytes to respond to anabolic factors during aging might play a role in the development of the age-related disease OA.

## Abbreviations

IL-1 = interleukin-1; LAP = latency associated peptide; OA = osteoarthritis; PBS = phosphate buffered saline; PG = proteoglycan; TGF-beta = transforming growth factor-beta; TGF-betaR = transforming growth factor-beta receptor.

## Competing interests

The authors declare that they have no competing interests.

## Authors' contributions

ENBD participated in the animal experiments and immunohistochemistry, carried out histological measurements, analyzed the data and drafted the manuscript. AS participated in the animal experiments, immunohistochemistry and analysis of the young versus old mice comparison. ELV participated in the animal experiments, carried out histological processing of the knee joints, participated in immunohistochemistry and performed ^35^S-sulfate measurements. PMK conceived of the study, participated in the design and coordination and helped to draft the manuscript. WBB participated in study design and revision of the final manuscript.
